# P-855. Influence of Prescription Medication Dispensing Kiosk Inventory Management and Antimicrobial Stewardship on Antibiotic Prescribing from Urgent Care Centers

**DOI:** 10.1093/ofid/ofaf695.1063

**Published:** 2026-01-11

**Authors:** Ethan Lobo, Corey M Frederick, Harrison Phan, Lauren Russell, Michael DeCoske, Yenny Ceballos, Ernesto Sanz Martinez, Timothy Gauthier

**Affiliations:** Baptist Health South Florida, Miami, Florida; Baptist Health South Florida, Miami, Florida; Baptist Health, West Covina, California; Baptist Health South Florida, Miami, Florida; Baptist Health South Florida, Miami, Florida; Baptist Health South Florida, Miami, Florida; Baptist Health South Florida, Miami, Florida; Baptist Health South Florida, Miami, Florida

## Abstract

**Background:**

In 2019, prescription medication dispensing kiosks were implemented within 20 Urgent Care Centers (UCCs) to enhance patient convenience and access to commonly prescribed medications, including several oral antibiotics. Later that same year, an outpatient antimicrobial stewardship program (ASP) was established to promote optimal antimicrobial use through education and policy interventions. This study aims to investigate the cumulative impact of ASP interventions, including continuous kiosk inventory management, on outpatient electronic prescriptions (eRx) for antibiotics from UCCs.

**Table 1:**
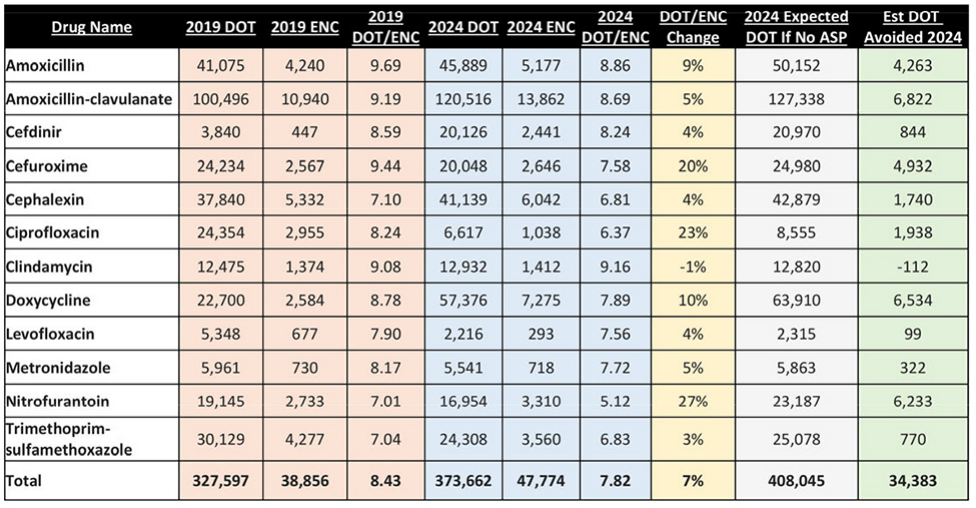
Prescribing outcomes, by antibiotic and overall ASP = antimicrobial stewardship program, DOT = days of therapy, ENC = encounter, EST = estimated

**Table 2:**
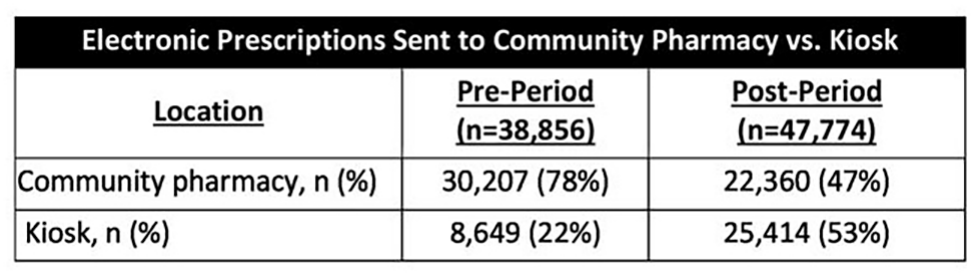
Prescriptions sent to kiosks versus community pharmacies

**Methods:**

A retrospective quality improvement project was undertaken to compare antibiotic eRx from UCCs in January - June 2019 versus January - June 2024. A variety of ASP interventions were implemented between the pre- and post-periods, including kiosk inventory updates and provider education. 12 antibiotics were selected for inclusion. The primary outcome of this analysis was antibiotic days of therapy (DOT) per eRx. Secondary outcomes included DOT volume by drug, eRx volume by drug, DOT/eRx by drug, and eRx routed to a kiosk versus community pharmacy. The impact of ASP interventions was estimated by calculating the difference in actual 2024 DOT/eRx versus what 2024 DOT would have been at the 2019 DOT/eRx rate.

**Results:**

During the pre-period, there were 243,684 global UCC encounters of which 38,856 (15.9%) had a target antibiotic prescribed. During the post-period, there were 284,038 global UCC encounters of which 47,774 (16.8%) had a target antibiotic prescribed. During the pre-period, a total of 327,597 DOT were prescribed, while during the post-period, 373,662 DOT were prescribed (8.43 DOT/eRx versus 7.82 DOT/eRx, p = 0.0058). Considering the prescribing patterns in 2019 versus 2024, it is estimated that nearly 35,000 DOT were avoided in the post-period. Encounter and DOT data at the antibiotic-level and overall are provided in Table 1. Data on antibiotic eRx filled through the kiosks is presented in Table 2.

**Conclusion:**

ASP engagement to optimize antibiotic eRx practices in UCCs contributed to a meaningful reduction in antibiotic DOT entering the community, which coincided with increased kiosk use and shorter durations of therapy.

**Disclosures:**

All Authors: No reported disclosures

